# Association between *FGA* gene polymorphisms and coronary artery lesion in Kawasaki disease

**DOI:** 10.3389/fmed.2023.1193303

**Published:** 2023-07-27

**Authors:** Xingzhu Liu, Yanfei Chen, Yanfei Yang, Zhongjian Su, Feng Wang, Chenghao Zhanghuang, Yuqin Wu, Xing Zhang

**Affiliations:** ^1^Department of Special Needs Ward, Kunming Children’s Hospital, Kunming, Yunnan, China; ^2^Department of Cardiology, Kunming Children’s Hospital, Kunming, Yunnan, China; ^3^Institute of Medical Biology, Chinese Academy of Medical Sciences, Kunming, Yunnan, China; ^4^Department of Urology, Yunnan Key Laboratory of Children's Major Disease Research, Kunming Children’s Hospital, Yunnan Province Clinical Research Center for Children’s Health and Disease, Kunming, Yunnan, China

**Keywords:** Kawasaki disease, coronary artery lesion, MMP, *FGA* gene, polymorphism

## Abstract

**Objective:**

To investigate the correlation between *FGA* gene polymorphisms and coronary artery lesion in Kawasaki disease.

**Methods:**

Two hundred and thirty four children with Kawasaki disease (KD group), 200 healthy children (normal group) and 208 children with non-KD fever (fever group) were enrolled. General clinical indicators, the concentration of serum MMPs, TIMP-1, FG-α,fibrinogen level, molecular function (FMPV/ODmax) and *FGA* Thr312Ala polymorphism were detected individually by testing peripheral venous blood after fasting in the morning.

**Results:**

There was no significant difference in average age among the three groups, which were 3.03 ± 1.22 years, 3.17 ± 1.30 years, and 3.21 ± 1.31 years, respectively. Compared with those in the fever group, the levels of white blood cell count (WBC), platelet count (PLT), procalcitonin (PCT), C-reactive protein (CRP), erythrocyte sedimentation rate (ESR), interleukin-6 (IL-6), monocyte chemoattractant protein-1 (MCP-1), and fibrinogen (Fg) levels were significantly increased in the KD group. Red blood cell count (RBC) and hemoglobin (Hb) levels were significantly decreased (*p* < 0.05).The concentration of serum MMPs, TIMP-1, and FG-α in the KD and fever groups were significantly higher than those in the normal group (*p* < 0.05). The concentration of MMP-2, MMP-3, MMP-9, MMP-13, TIMP-1, and FG-α in the KD group were significantly higher than those in the fever group (*p* < 0.05).The KD group was divided into two subgroups,55 patients with combined CAL and 179 patients without combined CAL. The plasma fibrinogen concentration in the combined CAL group was significantly higher than that in the non-combined CAL and normal groups (*p* < 0.01). There was no statistically significant difference in FMPV/ODmax among the three groups (*p* > 0.05). Compared with normal group, the FGA GG, GA, and AA genotype and G, A allele frequency of the FGA gene polymorphism in the KD group showed no significant difference (*p* > 0.05). In the KD group, the most common type in children with CAL was GA, while the most common type in children without CAL was GG.

**Conclusion:**

MMPs and FG-α were significantly upregulated in KD patients. The proportion of *FGA* genotype GA in children with CAL was significantly higher than that in children without CAL, suggesting that *FGA* gene polymorphisms affect coronary artery lesion in children with KD.

## Introduction

Kawasaki disease (KD) is a common systemic vasculitis disease prone to occur in children aged 6 months to 5 years. The main pathological changes of KD are systemic small–and medium-sized vasculitis, especially coronary arteritis. In severe cases, coronary artery lesion (CAL) and coronary artery aneurysm (CAA) may occur, which may lead to irreversible destruction of the vascular wall (1). Even after standardized treatment (high-dose gamma globulin + high-dose aspirin), 20% of children with KD still present with coronary artery disease, which can progress to ischemic heart disease (2, 3).

Plasma fibrinogen (FG) is an important protein involved in the process of coagulation and hemostasis, and its elevation is an independent risk factor for cardiovascular disease. In a prospective meta-analysis, it was found that every 1 g/L increase in *FGB* (β-148C/T-455G/A genotype)would double the mortality associated with coronary heart disease and stroke (4). FG consists of three polypeptide chains (α, β, γ), and their corresponding coding genes are *FGA*, *FGB*, and *FGG* (5). Previous studies have found that the *FGB* genotype affects FG levels and is a risk factor affecting intimal artery thickness (IMT) (6, 7); however, there are few reports on whether the *FGA* Thr312Ala genotype participates in the pathophysiological process of KD. In this study, we examined the levels of fibrinogen α (FGα) and total fibrinogen level in the peripheral venous blood and *FGA* genotype and analyzed the correlation between *FGA* Thr312Ala genetic polymorphism and coronary artery disease in KD to provide a theoretical basis for exploring a new treatment plan for KD.

### Data and methods

#### Subjects

This study included 234 children with KD, who were admitted to our hospital between January 2020 and October 2022 (KD group), 200 healthy children (normal group), and 208 non-KD children with fever (fever group) as the control group.

#### Inclusion and exclusion criteria

KD was confirmed according to the 2017 American Heart Association criteria (8). The study was approved by the medical ethics committee of our hospital. Written and signed informed consent was obtained from the parents/guardians of the children. The exclusion criteria included the use of glucocorticoids, immunosuppressants, and gamma globulin in the past 2 weeks; complicated with cardiovascular and respiratory diseases; incomplete clinical data or history of transfer during treatment; and unwillingness to cooperate with treatment.

The diagnostic criteria for coronary artery disease were as follows: Z-score was used as the diagnostic criteria for coronary artery disease. (1) Normal: *Z*-value <2; (2) Coronary artery dilation: 2 ≤ Z-value <2.5; (3) Small coronary aneurysm: 2.5 ≤ Z-value <5; (4) Medium coronary aneurysm: 5 ≤ Z-value <10, absolute diameter < 8 mm; (5) Giant coronary artery aneurysm: Z-value ≥10, or internal diameter > 8 mm. A Z-value ≥2.0 was considered CAL+.

### Research methods and indicators

#### Specimen collection

Five mL of peripheral venous blood was collected from each participant in the KD, normal, and fever groups after fasting in the morning on day 2 and day 10 after admission. Blood samples were centrifuged at 3000 rpm for 30 min. The supernatant was collected and placed in a PC box at −75°C. Each sample was split into two, one to test for general clinical indicators {MMPs, TIMP-1, FGα expression, and fibrinogen level and molecular function [fibrinogen monomer polymerization velocity (FMPV)/maximum optical density (ODmax)]μL} and the other for determining the *FGA* genotype.

#### DNA extraction

Take the PureGene DNA extraction kit and follow the instructions in the kit to extract the DNA of blood genosets. The specific steps are as follows: (1) 900uL blood cell lysate was added into Eppendorf tube; (2) Add 300uL of whole blood into the upper tube and mix it upside down. Incubate it at room temperature for about 10 min to make the red blood cells crack. During incubating, mix it upside down at least once; (3) Centrifuge at 12000xg at room temperature for 60s, then absorb and discard most of the supernatant with the suction head; (4) Oscillate the upper tube on the vortex to make the cells suspended in the residual fluid, so as to facilitate the next step of leukocyte lysis: (5) Add 450pL leukocyte lysis fluid into the hand tube and pump it repeatedly with the suction head to make the cell lysis: (6) At room temperature, 150uL of protein precipitating liquid was added into the upper tube, and then the solution was mixed by high speed oscillation with a vortex for 30s-60s; (7) Centrifuged at 4°C 12000xg for 3 min, the precipitated protein was a dense, dark brown substance; (8) Put the supernatant containing DNA into a new Eppendorf tube, and then add 600uL 100% isopropyl alcohol, and gently pour 50 times to make the solution mixed; (9) After centrifugation at 4°C 12000xg for 1 min, white DNA precipitates can be seen; (10) Run to the superclear liquid, on the drying paper short dry suction pipe in the remaining liquid, then add 70% ethanol 450uL, and gently up and down frequency countdown, to wash the DNA precipitation: (11) at 12000xg high heart 2 min, carefully pour away the ethanol; (12) Place the centrifugal tube upside down on the drying paper and dry it in air for 10 min to 15 min; (13) The DNA bath solution wave was added into the upper tube, incubated at 65°C for 1 h, and the Eppendorf tube was flipped to promote DNA dissolution.

### Research methods

#### FMPV/ODmax

10 μL reaction mixture and 50 μL plasma were fully mixed in a 0.5 cm optical path colorimetric cup and immediately placed into the spectrophotometer color chamber. The fibrinogen monomer polymerization process was detected at 340 nm, and FMPV and ODmax were determined. Then, the FMPV/ODmax was calculated to reflect the molecular function of fibrinogen.

#### ELISA

ELISA kits of MMP-2, MMP-3, MMP-9, MMP-13, TIMP-1, and FG-α were used. Samples were added according to the layout of the plate. A total of 50 μL sample (10 μL sample and 40 μL sample diluent; a 5-fold dilution) or 50 μL standard was added per well. Then, 100 μL horseradish peroxidase labeled detection antibody was added to each well, and the plate was sealed with a plate membrane and incubated at 30°C for 60 min. After which, the liquid was discarded, 200 μL detergent was added per well, and the plate was left to stand for 1 min on an absorbent paper. Then, the detergent was discarded, the plate was pat dry on the absorbent paper, and this process was repeated five times. Next, 50 μL of soybean substrate A and B were added to each well and the plate was incubated for 15 min at 30°C in the dark. Lastly, 50 μL of termination solution was added to each well and the absorbance value of each well was measured at a wavelength of 450 nm.

DNA extraction and polymorphism detection: White blood cells were separated from venous blood using the 2% EDTA hypotonic method. DNA was extracted with phenol:chloroform:isoamyl alcohol (25,24:1) and precipitated with ethanol. The target DNA was amplified by polymerase chain reaction (PCR). The upstream primer was 5 ‘- GGAGTGGAAGGCATTAACAGA-3’, and the downstream primer was 5 ‘- GGGTTTTGGTTTTTCCAGTACTTC-3’. The total reaction volume was 30 μL. The reaction mixture contained DNA 0.3 μg, 0.2 mM dNTPs, 10 pM primers, 1.5 mM MgCl_2_, and 1 U Taq DNA polymerase. The PCR conditions were as follows: 95°C for 3 min predegeneration, (95°C for 20 s, 63°C for 30 s, 72°C for 1 min) × 28 cycles, and 72°C for 7 min. Then, 10 μL of the PCR product was added to 10 U of endo Rse I, incubated at 37°C overnight, and subjected to electrophoresis with 2% agarose.

#### Statistical methods

SPSS 22.0 was used for statistical analysis, and the Mann–Whitney U test was used for comparison of differences between groups. One-way analysis of variance (ANOVA) and the Tukey test were used for comparison of multiple groups, followed by the LSD method for comparison between two groups. *p* < 0.05 was considered statistically significant.

## Results

Comparison of general clinical indicators: There was no significant difference in average age among the three groups, which were 3.03 ± 1.22 years, 3.17 ± 1.30 years, and 3.21 ± 1.31 years, respectively. Compared with the normal group, the fever time in Kawasaki disease group and fever group was significantly prolonged. The levels of white blood cell count (WBC), red blood cell count (RBC), hemoglobin, platelet, procalcitonin (PCT), C-reactive protein (CRP), erythrocyte sedimentation rate (ESR), interleukin-6 (IL-6), monocyte chemotactic protein-1 (MCP-1) and fibrinogen (Fg) were significantly increased. The level of above indexes in Kawasaki disease group was higher than that in fever group, and the difference was statistically significant (*p* < 0.05; Table 1).

**Table 1:** Comparison of general clinical indicators.

	Normal group (*n* = 200)	KD group (*n* = 234)	Fever group (*n* = 208)	*P*
Age(y)	3.03 ± 1.22	3.17 ± 1.30	3.21 ± 1.31	0.227
Gender(male)	89(44.50)	107(45.73)	92(44.23)	0.558
Fever time(d)	0.00 ± 0.00	4.25 ± 1.33	1.93 ± 0.79	<0.001
WBC(10^3^/μL)	8.41 ± 2.65	17.22 ± 5.44^*^	11.53 ± 3.86	<0.001
RBC(10^6^/mm3)	4.79 ± 0.64	3.66 ± 0.39^*^	4.26 ± 0.49	<0.001
Hb(g/dL)	11.83 ± 1.32	9.81 ± 1.19^*^	11.47 ± 1.26	<0.001
PLT(10^3^/μL)	310.66 ± 108.18	411.53 ± 139.22^*^	344.92 ± 117.84	<0.001
CRP(mg/dL)	10.11 ± 5.92	58.29 ± 29.48^*^	26.43 ± 13.44	<0.001
PCT(ng/L)	0.43 ± 0.22	1.21 ± 0.88^*^	0.68 ± 0.26	<0.001
ESR(mm/h)	3.03 ± 1.22	65.97 ± 30.52^*^	3.03 ± 1.22	<0.001
IL-6(pg/ml)	13.25 ± 6.22	47.03 ± 20.22^*^	35.44 ± 19.85	<0.001
MCP-1(pg/ml)	104.55 ± 37.22	222.47 ± 67.30^*^	149.36 ± 48.36	<0.001
Fg(g/L)	3.14 ± 0.68	8.83 ± 1.22^*^	5.26 ± 0.96	<0.001

WBC, white blood cell count; RBC, red blood cell count; Hb, hemoglobin; PLT, platelet count; CRP, C-reactive protein; PCT, procalcitonin; ESR, erythrocyte sedimentation rate; IL-6, interleukin-6; MCP-1, monocyte chemoattractant protein-1; Fg, fibrinogen. *The statistically significant (*p*-values < 0.05)compared with those in the fever group.

The concentration of serum MMPs, TIMP-1, and FG-α: The concentration of serum MMPs, TIMP-1, and FG-α in the KD and fever groups were significantly higher than those in the normal group (*p* < 0.05). The concentration of MMP-2, MMP-3, MMP-9, MMP-13, TIMP-1, and FG-α in the KD group were significantly higher than those in the fever group (*p* < 0.05; Table 2).

**Table 2:** The expression levels of serum MMPs, TIMP-1, and FG-α.

	Normal group (ng/mL)	KD group (ng/mL)	Fever group (ng/mL)	*F*	*P*
TIMP-1	105.699 ± 6.525	188.476 ± 14.064^*#^	136.842 ± 12.573^*^	68.3	< 0.0001
MMP-2	3.087 ± 0.483	4.494 ± 0.27^*#^	3.877 ± 0.574^*^	13	0.0004
MMP-3	38.65 ± 5.596	65.302 ± 4.576^*#^	49.161 ± 4.684^*^	46.3	< 0.0001
MMP-9	75.124 ± 6.439	115.262 ± 10.844^*#^	94.482 ± 3.804^*^	36.94	< 0.0001
MMP-13	38.576 ± 19.621	91.793 ± 33.871^*#^	66.84 ± 6.935^*^	6.964	0.0062
FG-α	754.585 ± 184.877	981.017 ± 347.37^*#^	835.64 ± 265.378^*^	4.941	0.037

*The statistically significant (*p*-values < 0.05)compared with those in the normal group; #The statistically significant (*p*-values < 0.05)compared with those in the fever group.

Comparison of fibrinogen and its function: The KD group was further divided into two subgroups; 55 patients with combined CAL and 179 patients without combined CAL. The results showed that the plasma fibrinogen concentration in the combined CAL group was significantly higher than that in the non-combined CAL and normal groups (*p* < 0.01). There was no statistically significant difference in FMPV/ODmax among the three groups (*p* > 0.05; Table 3).

**Table 3:** Comparison of fibrinogen and its function.

Group	*N*	Fibrinogen (g /L)	FMPV/ODmax
Normal group	200	3.14 ± 0.68	3.54 ± 0.63
KD group	234		
CAL subgroup	55	9.52 ± 1.06*	3.91 ± 0.46
No CAL subgroup	179	6.23 ± 0.83	3.70 ± 0.54
*F*		9.773	0.779
*P*		<0.01	>0.05

*The statistically significant (*p*-values < 0.05)compared with those in the normal group and no CAL subgroup.

*FGA* gene polymorphism: Compared with normal group, the FGA GG, GA, and AA genotype and G, A allele frequency of the FGA gene polymorphism in the KD group showed no significant difference (*p* > 0.05). In the KD group, the most common genotype in children with CAL was GA, while the most common genotype in children without CAL was GG.There was a statistical difference between the two groups(*p* < 0.05; Table 4).

**Table 4:** *FGA* gene polymorphism and the allele distribution frequency.

Group	*N*	Gene polymorphism (%)			The allele distribution frequency (%)	
		GG	AA	GA	G	A
KD group	234	127 (54.3)	22 (9.4)	85 (36.3)	339 (72.4)	129 (27.6)
Normal group	200	102 (51.0)	20 (10.0)	78 (39.0)	282 (70.5)	118 (29.5)
*X*^2^		1.221	0.885			
*P*		>0.05	>0.05			
KD group						
CAL subgroup	55	23 (41.8)	6 (10.9)	26 (47.3)	37 (67.3)	18 (32.7)
No CAL subgroup	179	127 (70.9)	30 (16.8)	22 (12.3)	138 (77.1)	41 (22.9)
*X*^2^		8.771	0.886			
*P*		<0.05	>0.05			

*The statistically significant (*p*-values < 0.05)compared with those in the no CAL subgroup; #The statistically significant (*p*-values < 0.05)compared with those in the CAL subgroup.

## Discussion

KD is characterized by the abnormal activation of the immune system and extensive damage to the endothelial system. In the acute phase of KD, the abnormal activation of the immune system leads to the production of inflammatory mediators (proteases and reactive oxygen species), which is believed to induce pathological changes in the vascular system (9). Children with KD that do not undergo standardized treatment are prone to CALs (10), which can lead to myocardial infarction, coronary artery dilation, and coronary artery aneurysm (CAA). Although clinical treatment of KD has made progress, 20% of children with KD children still progress to CAL. An autopsy study found that the coronary artery was the most severely damaged part of the intima; other parts included the aorta, abdominal aorta, carotid artery, subclavian artery, and pulmonary artery (11). Pathological changes are similar to those of nodular polyarteritis in infants, including thickening of the arterial intima, invasion of granulocytes and monocytes, rupture of the inner elastic fiber layer and medial membrane, necrosis of the vascular wall, and formation of aneurysms (12, 13).

This study found that the serum levels of MMPs in children with KD were significantly increased. It is worth noting that MMPs are markers of inflammation. Therefore, the elevated concentration of matrix metalloproteinases in circulation may be a manifestation of coronary artery inflammation. For example, elevated levels of circulating MMPs have also been observed in other inflammatory diseases (such as pneumonia or septicemia), and these diseases do not cause CAL (14); therefore, it is suggested that significantly elevated levels of MMPs play an important role in the destruction of the coronary artery wall, resulting in aneurysms (15). Several studies have compared the MMP levels in KD with other febrile or inflammatory diseases, including bronchitis, pneumonia, sepsis, gastroenteritis, and encephalitis. Previous studies have consistently shown that although CRP shows similar levels of inflammation, the level of MMPs in KD, especially MMP-9, is much higher than that in other inflammatory diseases (about 4–8 times) (16, 17). In addition, MMP levels in pneumonia are positively correlated with CRP levels, but this correlation is not as clear in KD because MMP levels in patients with CALs are significantly higher than in non-CAL patients (18). In another study, the levels of MMPs and TIMPs in febrile non-KD patients were significantly higher than those in the non-febrile healthy control group;however, there was no significant difference in the MMP/TIMP ratio between febrile patients and the non-febrile control group, showing that the normal MMP/TIMP ratio was maintained in inflammatory diseases that did not cause CAAs (19). Only patients with KD showed an imbalance between MMPs and TIMPs, which tended to increase the activation of MMPs. In addition, the interaction between MMPs, MMPs and TIMPs and MMPs and fibrinogen may affect their activity and regulate the pathophysiological process of arterial wall destruction. Existing evidence shows that genetic factors contribute to the formation of adult aneurysms (20). *MMP-9* gene promoter polymorphism has been found in patients with intracranial aneurysm or myocardial infarction. The variation in this polymorphism leads to variation in transcription levels. It has been reported that the polymorphisms of *MMP-3* promoter and *TIMP-1* gene are related to abdominal aortic aneurysm (21). Therefore, in addition to MMPs and TIMPs, we speculate that genetic factors may be involved in the formation of KD aneurysms.

This study found that blood hypercoagulability continues to exist in children with KD from the acute phase until the next few months or years, and there is a possible correlation with the occurrence of CALs (22). Wu MH^，^S study findings (23) estimated the overall prevalence of KD (≈1/2940) in a population < 40 years. They, particularly the males, carry long-term coronary risks from a young age. Risk stratification for a timely coronary intervention and risk modification are mandatory.Lee JJY^，^S study (24) evaluated the risk of hypertension, major adverse cardiac events (MACE), and all-cause mortality in patients with KD until young adulthood.KD patients with coronary artery aneurysms have a higher risk of developing cardiovascular disease.Blood hypercoagulability is related to abnormal vascular endothelial function, hemorheology, and blood components (including platelets, coagulation, and fibrinolysis). Both the acute and recovery phases of KD are accompanied by abnormalities in vascular endothelial function. Abnormalities in platelet activation and fibrinolysis continue to exist in the acute phase of KD and can persist for a long time after the disease. The polymorphism of Thr312Ala of fibrinogen Aα chain may be associated with PTE, in which GG genotype significantly increases the risk of PTE, and G allele may be the genetic factor associated with PTE pathogenesis. TIMPs-MMPs complex is widely involved in vascular wall inflammation. Children with immune vascular injury, endothelial cell damage, subendothelial matrix exposure, platelet adhesion, aggregation, activation, and release of various inflammatory mediators, thus activating the coagulation pathway and affecting the inflammatory process (25). Vascular inflammation can promote the occurrence of blood hypercoagulability, and the activation factors produced in the coagulation process can also affect the inflammatory process. Fibrinogen is an important cofactor involved in platelet activation, inducing platelet aggregation, increasing blood viscosity, and promoting thrombosis (26). Abnormal blood flow exists in the coronary arteries of children with KD and CALs, which becomes slow or even stagnant owing to the reduction of shear stress. These findings suggest that the occurrence of CALs in children with KD is related to an increase of fibrinogen (27).

The gene encoding fibrinogen is located on the long arm of chromosome 4, and the three polypeptide chains are encoded by the *FGA*, *FGB*, and *FGG* genes. Gene transcription of fibrinogen is coordinated; any kind of mRNA transcription can promote the transcription of other mRNA, thus increasing the synthesis and secretion of molecular fibrinogen (28). Previous studies have found that multiple sites of the *FGB* gene may be related to fibrinogen expression (3), which are also genetic risk factors related to coronary heart disease. However, the relationship between the *FGA* gene of the polypeptide chain and the occurrence of CALs is still unclear, but it was found that expression levels of *FGA* in children with KD complicated with CALs were significantly higher than in the normal and fever groups. This suggests that it might participate in the development of CALs. Further exploration found that *FGA* can affect the interaction between nuclear protein and IL-6 inflammatory factor components, thus affecting the expression level of fibrinogen (29). The change in *FGA* polymorphism may increase the affinity of nuclear transcription factors with corresponding regulatory sites, thus further enhancing gene transcription and affecting the development of thrombosis tendency (30).

*FGA* polymorphisms are closely associated with adult coronary artery disease, and CAL is the most serious complication of KD. However, there are few studies on the relationship between *FGA* gene polymorphisms and CAL in KD. This study observed the *FGA* genotype and allele frequency, fibrinogen level, and molecular function changes in children with KD and CAL, children without CAL, and normal children. It was found that the KD with CAL GA genotype was significantly higher than in normal children, and fibrinogen levels were significantly higher than those in the children without CAL and normal groups. Therefore, it was speculated that *FGA* gene polymorphisms might be related to the pathogenesis of KD, and individuals carrying the GA genotype may have an increased risk of CAL by affecting fibrinogen levels; therefore, it is important to detect fibrinogen levels and determine the *FGA* genotype in clinical practice for early prediction of the risk of CAL in children with KD.

## Conclusion

In summary, MMPs and FG-α were significantly upregulated in KD patients. The proportion of *FGA* genotype GA in children with CAL was significantly higher than that in children without CAL, suggesting that *FGA* gene polymorphisms affect coronary artery disease in children with KD.

## Data availability statement

The raw data supporting the conclusions of this article will be made available by the authors, without undue reservation.

## Ethics statement

This study was approved by the ethical committee of Kunming Children’s Hospital (2022-03-144-K01). Written informed consent to participate in this study was provided by the participants’ legal guardian/next of kin. Written informed consent was obtained from the individual(s), and minor(s)’ legal guardian/next of kin, for the publication of any potentially identifiable images or data included in this article.

## Author contributions

XL, YC, YW, CZ, and XZ carried out the studies, collected the data, and drafted the manuscript. YY and ZS performed the statistical analysis and participated in its design. CZ, ZS, and FW helped to draft the manuscript. All authors read and approved the final manuscript.

## Funding

This study was supported by Kunming “Spring City Plan” High-level talent introduced by the Engineering Young Talents Special Project, Technology Talent “1000” training Project [No. 2022- SW (Reserve)-0014], Yunnan Education Department of Science Research Fund (No. 2023 J0295), Department of Science and Technology of Yunnan Province Kunming Medical University Joint Project (No. 202301AY070001-108), Kunming City Health Science and Technology Talent “1000” training Project [No. 2020- SW (Reserve)-112], Kunming Medical Joint Project of Yunnan Science and Technology Department (No. 202001 AY070001-271), and Open Research Fund of Clinical Research Center for Children’s Health and Diseases of Yunnan Province (2022-ETYY-YJ-03). The funding bodies played no role in the study’s design and collection, analysis and interpretation of data, and writing the manuscript.

## Conflict of interest

The authors declare that the research was conducted in the absence of any commercial or financial relationships that could be construed as a potential conflict of interest.

## Publisher’s note

All claims expressed in this article are solely those of the authors and do not necessarily represent those of their affiliated organizations, or those of the publisher, the editors and the reviewers. Any product that may be evaluated in this article, or claim that may be made by its manufacturer, is not guaranteed or endorsed by the publisher.
